# Recent advances in HIV-associated Kaposi sarcoma

**DOI:** 10.12688/f1000research.17401.1

**Published:** 2019-06-26

**Authors:** Alessia Dalla Pria, David J. Pinato, Margherita Bracchi, Mark Bower

**Affiliations:** 1 Imperial College London, London, UK; 2 Chelsea and Westminster Hospital, London, UK

**Keywords:** KS, cART, HIV

## Abstract

Kaposi sarcoma (KS) is a mesenchymal tumour caused by KS-associated herpesvirus and is an AIDS-defining illness. Despite a decline in incidence since the introduction of combination anti-retroviral therapy, KS remains the most common cancer in people living with HIV in sub-Saharan Africa, where it causes significant morbidity and mortality. This review reflects on recent epidemiological data as well as current management, unmet needs and future perspectives in the treatment of HIV-associated KS with particular emphasis on the potential role of immune checkpoint inhibitors.

## Introduction and context

Kaposi sarcoma (KS) is an angio-proliferative disorder that was first described in its classic form by the Hungarian physician Moritz Kaposi in 1872. It is categorised in different disease types that are all aetiologically associated with KS-associated herpesvirus (KSHV), also known as human herpesvirus 8 (HHV-8). The malignant cell of KS is the spindle cell which is infected by KSHV and is believed to be of lymphatic endothelial origin. KS lesions are composed of a combination of abnormal vascularity, inflammation and fibrosis with hemosiderin deposits.

There are four widely recognised epidemiological forms of KS: classic KS, which is a relatively indolent disease that mainly affects elderly men from the Mediterranean basin and Middle East; African (endemic) KS, which pre-existed the HIV/AIDS pandemic ^1^; allograft transplantation-associated (iatrogenic) KS; and AIDS-associated (epidemic) KS, which is characterised by potentially aggressive clinical features.

Despite being a relatively infrequent malignancy worldwide, KS remains the most common cancer in people living with HIV (PLWH) in the African continent and is one of the most common tumours, especially in children in central, eastern and southern Africa ^2, 3^. WHO-GLOBOCAN data indicate that there were 41,799 new KS diagnoses (28,248 males and 13,551 females) and 19,902 (13,117 males and 6,785 females) KS deaths globally in 2018 ^4^.

The introduction of combination anti-retroviral therapy (cART) led to a remarkable decline in AIDS-KS incidence ^5^ and to a significant improvement in KS prognosis ^6, 7^. However, KS still accounts for significant morbidity and mortality, particularly in sub-Saharan countries. Although the incidence of KS fell with the cART-induced rise in CD4 T-cell count and immune reconstitution, the risk of developing KS in PLWH with a normal CD4 T-cell count remains substantially higher than in the general population ^8^.

## Management of HIV-KS

A conspicuous body of clinical evidence indicates that AIDS-KS can regress following starting cART alone ^9, 10^, suggesting a proportion of cases to be fully sensitive to cART-mediated immune reconstitution. After starting cART, patients with anti-retroviral–naïve HIV recover their immunity, at least in part, within months. Responses to cART alone are variable. Complete remission of KS occurs in 20 to 80% of patients and is more frequent in cART-naïve patients with limited-stage disease who are compliant with HIV therapy ^11^. The mechanism of KS regression is thought to be immunological and specifically associated with reconstitution of an effective cytotoxic cell–mediated response targeting KSHV. As KS can rapidly progress and affect vital organs, patients with advanced-stage KS ( Table 1) need additional systemic anti-cancer treatment alongside cART ^12^. Additionally, a proportion of HIV-infected patients will develop KS *de novo* or will experience disease progression after starting cART and this phenomenon may be an indicator of unmasking and paradoxical immune reconstitution inflammatory syndrome. There are no controlled trials to evaluate the best care of such patients, although systemic chemotherapy for KS, and not steroid treatment that is associated with disease progression, seems appropriate as reported in small series and case reports ^13– 16^.

**Table 1.** Kaposi sarcoma staging.

Kaposi sarcoma staging	Early stage/good risk (all of the following)	Advanced stage/poor risk (any of the following)
T (tumour)	T0: Confined to skin, lymph nodes or minimal oral disease	T1: Tumour-associated oedema Ulceration Extensive oral disease Visceral disease (non- nodal)
I (immune status)	I0: CD4 >150/mL	I1: CD4 <150/mL

Prior to the advent of cART, a number of agents, including interferon-alpha, thalidomide, retinoids and cytotoxics such as vincristine and bleomycin, were used in the treatment of HIV-KS. However, the advent of liposomal anthracyclines in the early 1990s is recognised as a significant advancement in the chemotherapy of KS and has become the gold standard of care. Their role has been consolidated by level-I evidence from three randomised controlled trials that showed better response rates and favourable toxicity profiles ^17, 18^ compared with previous standard chemotherapy of bleomycin and vincristine with or without Adriamycin (doxorubicin). Treatment responses were often non-sustained in these initial trials that predated the introduction of cART. In advanced-stage KS, the concomitant use of cART and liposomal anthracyclines achieves a 70% overall response rate with prolonged remission ^19^. Over time, improvements in the understanding of kinetics of immune reconstitution and the consolidating knowledge that cART is an effective and sufficient therapy for people with early stage KS (T0) ^20^, but not for patients with advanced disease (T1 stage), led to the consolidation of a stage-stratified approach in patients with KS. This approach, proposed in the British HIV Association guidelines for HIV malignancies (BHIVA) ^21^, supports the use of cART alone for early-stage KS (T0), reserving the addition of liposomal anthracycline chemotherapy for more aggressive disease (T1), based on evidence of efficacy in PLWH with visceral KS. The effectiveness of a stage-stratified approach to KS was further consolidated by a landmark study that described a cohort of 469 patients, confirming that this approach achieves high survival rates in patients with advanced KS (5-year overall survival [OS] 85%) and reduces exposure to chemotherapy in patients with early-stage disease (5-year OS 95%) ^22^.

Even with the notable advancements in the systemic management of KS, up to 15% of patients develop progressive disease despite achieving good immune-virologic control of HIV with undetectable plasma HIV-1 RNA and adequate CD4 T-cell counts (>300/mm ^3^) ^23– 25^. The immunopathology of cART-refractory KS is complex. Mechanistic studies have recognised that this type of disease is associated with T cell–immune senescence as a key pathophysiologic trait and that in some patients it has an indolent clinical course similar to that of the classic subtype ^24, 26^. In our HIV-cancer centre, this group of patients usually receives multiple cycles of chemotherapy alternating between liposomal anthracycline and paclitaxel. Sequential alternation of non–cross-reacting chemotherapy agents has been found to be an effective approach to control HIV-KS in a series of 20 patients however, no comparative studies are available and this treatment strategy may lead to increased chemotherapy toxicities ^27^. Cardiotoxicity is linked to cumulative dosage of anthracyclines ^28^, although the risk is lower with the liposomal formulation and high cumulative dosages of taxanes, used as a second-line therapy, are associated with irreversible peripheral neuropathy ^29^.

Local treatments, such as surgical excision, intralesional vinblastine and radiotherapy, are rarely used as they do not have an impact on the natural history of the systemic disease and specifically radiation therapy can be associated with long-lasting local toxicity. They can be used to control non-rapidly progressive cutaneous lesions where cART alone has not achieved a good result. Intralesional vinblastine is administered for lesions that are smaller than 1 cm whereas radiotherapy may be used for bigger lesions (>1 cm).

Although recent efforts have made cART available in most resource-limited settings, access to chemotherapy remains a major problem. More affordable chemotherapy agents such as (non-liposomal) doxorubicin, bleomycin, vincristine and etoposide may be currently administered in sub-Saharan Africa but they are associated with increased toxicity and lower efficacy for HIV-associated KS. Moreover, the infrastructure and expertise necessary to safely reconstitute and deliver intravenous chemotherapy are not available in many hospitals in Africa ^30, 31^. This is one of the main unmet needs in the management of KS in resource-poor countries and research and development as well as health policy efforts should therefore focus on the availability of oral, easily delivered, and inexpensive regimens in these regions.

A recent study conducted by the AIDS Clinical Trials Group and the AIDS Malignancy Consortium in Africa and Brazil demonstrated that oral etoposide is inferior to both intravenous paclitaxel and intravenous combination bleomycin and vincristine in advanced disease and that paclitaxel is somewhat better than combination bleomycin and vincristine ^32^.

## Recent advances

It must be noted that there is no accepted animal model for KS or KSHV infection for testing of drug therapies prior to clinical trials. Hence, in recent years, multiple targeted therapies for KS, especially oral medicines, have been directly routed to human trials. The most promising results have been achieved with pomalidomide, an orally available derivative of thalidomide that is less neurotoxic and has immune-modulatory, anti-angiogenic, and anti-proliferative activities. The effects against KS include modulation of tumour necrosis factor-alpha, interleukin-6, and vascular epithelial growth factor (VEGF) and enhancement of CD4 ^+^ and CD8 ^+^ T-cell co-stimulation. Notably, it is logically presumed with limited supporting *in vivo* data that the latter immunological factors are critical drivers in the development of KS ^33^. In a phase 2 study, 22 patients who received treatment achieved an overall response rate of 73%. Responses were rapid and occurred in HIV-positive and HIV-negative patients with advanced KS and in heavily pre-treated subjects ^34^. A trial conducted with a similar immunomodulatory agent, lenalidomide, had somewhat less favourable results with a response rate of 40% using the AIDS Clinical Trial Group (ACTG)-defined criteria and a 0% Physical Global Assessment (PGA) criteria response rate ^35^ at week 48. Uldrick *et al*. ^36^ published phase 2 trial results of the humanised monoclonal antibody bevacizumab, which targets VEGF-A, in HIV-associated KS. The overall response rate was only 31% despite the interesting role of VEGF-A in KS pathogenesis as a paracrine and autocrine growth factor for KS cells *in vitro* ^37^. This points to the need for *in vivo* experimental data to support a definitive role for VEGF-A in the development of KS. Disappointing results have also been obtained with matrix metalloproteinase inhibitors ^38^ and other angiogenesis inhibitors ^39^. Given that activation of platelet-derived growth factor (PDGF) and tyrosine kinase (TK) receptor c-kit has been demonstrated to have a role in KS pathogenesis both *in vitro* and *in vivo*, imatinib, a selective inhibitor of the TK Abl and c-kit, has been tested in HIV-KS with promising results ^40^. Similarly, KSHV-encoded proteins upregulate the mitogen-activated protein kinase (MAPK) pathway ^41^, and a clinical trial of the use of selumetinib, an inhibitor of this pathway, in HIV-KS is being completed. Rapamycin has been shown to be effective in transplantation-associated KS and has also been found to have activity in AIDS-KS but has significant drug–drug interactions with cART. Despite the pharmacokinetic interactions, that result in more than 200-fold differences in cumulative rapamycin doses between participants on protease inhibitor (PI)- and non-nucleoside reverse transcriptase inhibitor (NNRTI)-containing regimens, treatment was well tolerated ^42^.

The mechanisms behind the persistence or *de novo* development of HIV-associated KS in virally suppressed patients with a preserved CD4 T-cell count remain unclear. Anti-cancer immune response is closely linked to adaptive T-cell responses as part of a “cancer-immunity cycle” ^43^. This immune response is initiated with antigen priming of T lymphocytes: antigens are released by dying tumour cells and presented by dendritic cells via the major histocompatibility complex to the T-cell receptor. Primed T cells then traffic from the lymph node to the tumour site where they recognise and kill the cancer cells in the effector phase of adaptive immune response. Tumours have developed immune tolerogenic strategies that are delivered through complex mechanisms, including the modulation of multiple co-inhibitory and co-stimulatory pathways. Two immune checkpoints—cytotoxic T-lymphocyte–associated protein 4 (CTLA-4) and programmed cell death protein 1 (PD-1) pathways—respectively inhibit the priming and effector phase of cancer-specific immune response. They are therefore of great clinical interest in the development of new cancer immunotherapies.

In the context of chronic viral infections, including HIV and malignancy, the upregulation of PD-1 on cytotoxic T lymphocytes is a well-described mechanism of immune tolerance. In the HIV infection setting, chronic inflammation can lead to the exacerbation of immune exhaustion pathways with a subsequent decline in cancer immune surveillance and the development of a favourable oncogenic microenvironment ^44^. *In vitro* studies indicate that PD-L1 blockade reverses immune dysfunction of HIV-specific CD8 T cells with increased survival proliferation and cytokine production ^45^. Host *et al*. have also demonstrated that KSHV infection of primary human monocytes upregulates PD-L1 transcription and expression which highlight a new immune evasion strategy for KSHV ^46^.

In cART-refractory KS, our group has evaluated the potential for the PD-1 immune tolerance pathway as a mechanism underlying the pathogenesis and development of active KS in patients with well-controlled HIV stable on cART. Though limited by a small sample size of 10 patients, we confirmed PD-L1 upregulation with evidence of weak cytoplasmic PD-L1 expression in 50% of cases. Though differing from the intense membranous pattern of expression in epithelial malignancies that respond to PD-L1/PD-1 blockade *in vivo*, PD-L1–positive KS was associated with greater T-cell and macrophage infiltrate, hallmarks of an exhausted immune response to cancer ^47^.

Since our study, Galanina *et al*. reported on the efficacy and toxicity of immune checkpoint inhibitors (anti-PD-1 nivolumab and pembrolizumab) in nine patients with HIV-associated KS ^48^. The authors conducted a retrospective review of a heterogeneous group of patients: four patients had cutaneous disease only (T0), five patients had visceral disease (T1), seven out of nine patients had well-controlled HIV, and two had detectable plasma HIV RNA. Five patients had a good-risk immune status (I0) with a CD4 T-cell count above 200 cells/mL, and four patients had a CD4 count below the 200 cells/mL threshold ^48^. The patients were also heterogeneous in terms of prior treatment, having received from none up to four lines of systemic therapy prior to anti-PD-1 blockade, including liposomal anthracycline, paclitaxel, lenalidomide and bortezomib. One patient achieved complete remission, five out of nine patients achieved partial response, and three patients had stable disease. No one experienced KS progression and they were still on treatment at the time of reporting. Patients were evaluated for response every 4 weeks with KS response criteria defined by the AIDS Malignancy Consortium. The median progression-free survival has not been reached in the nine patients at a median follow-up of 5 months. No serious toxicity above grade 2 was observed in the study, and the most common side effects were fatigue, muscle aches and pruritus. Similar results were published in two cases of HIV-negative KS treated with nivolumab ^49^. The authors’ publication supports our provocative findings that PD-1 checkpoint blockade could have a therapeutic role in HIV-KS even in the presence of weak or no PD-L1 tumour expression in a similar way that good responses are observed in other virally induced tumours such as polyomavirus (MCPyV)-associated Merkel cell carcinoma ^50^. Nevertheless, the study is preliminary and does not lead to definitive conclusions as to the positioning of PD-1 therapy in KS. Longer prospective trials with the aim to better evaluate this treatment strategy are under way ^51^.

An additional benefit to the anti-tumour effect of anti-PD-1 therapy might be the potential role in HIV cure strategies. This immunotherapy is potentially able to reactivate latent HIV-1 and enhance HIV-specific antiviral response. The results on depleting latent HIV reservoirs so far are inconsistent ^52– 54^ and limited but the effect of immune checkpoint inhibitors in this setting remains an area of interest where prospective studies are warranted. Especially because immune checkpoint blockade is associated with a risk of life-threatening autoimmune pathologies ^55^, studies of PD-1 blockade as a curative strategy in HIV should proceed with caution. Recent data are also highlighting the role of the PD-1 pathway in the priming of HIV T-cell responses; therefore, extra consideration should be taken in the timing of blocking this signalling pathway in order to avoid potential negative effects on the induction of *de novo* T-cell responses ^56^. Benefits may be limited to specific groups of patients with currently undefined predictors of response. Given the favourable toxicity profile of PD-1 blockade in the study by Galanina *et al*., the PD1 blockade described is more justified when the HIV research is combined with a cancer treatment strategy such as in KS.

## Conclusions

The introduction of cART has not resolved the challenge of HIV-associated KS. Unmet needs include access to liposomal anthracycline chemotherapy in sub-Saharan Africa for patients with advanced disease and the development of new treatment strategies for patients with cART-refractory KS who are currently intermittently treated long term with potentially toxic chemotherapy. The immune checkpoint inhibitor certainly represents a very interesting area to explore further given the parallel potential as an HIV cure strategy.
